# Choline Kinase Emerges as a Promising Drug Target in Gram-Positive Bacteria

**DOI:** 10.3389/fmicb.2019.02146

**Published:** 2019-10-15

**Authors:** Tahl Zimmerman, Juan Carlos Lacal, Salam A. Ibrahim

**Affiliations:** ^1^Food Microbiology and Biotechnology Laboratory, Food and Nutritional Sciences Program, North Carolina Agricultural and Technical State University, Greensboro, NC, United States; ^2^Department of Oncology, Hospital Universitario de Fuenlabrada, Madrid, Spain

**Keywords:** choline kinase, antimicobial, antibiotic, gram-positive, *Streptococcus pneumoniae*

## Abstract

Both nosocomial pathogens, such as *Streptococcus pneumoniae* and *Haemophilus influenzae* and food-borne pathogens, such as *Bacillus cereus* and *Clostridium perfringens* are known to be detrimental to human and animal health. The effectiveness of currently used treatments for these pathogens becomes limited as resistant strains emerge. Therefore, new methods for eliminating bacterial pathogens must be developed continuously. This includes establishing novel targets to which drug discovery efforts could be focused. A promising method for discovering new drug targets in prokaryotes is to take advantage of the information available regarding the enzymatic pathways that have been established as drug targets in eukaryotic systems and explore the analogous pathways found in bacterial systems. This is an efficient strategy because the same inhibitors developed at considerable expense to block these pathways in eukaryotic systems could also be employed in prokaryotes. Drugs that are used to prevent diseases involving eukaryotic cells could be repurposed as antibiotics and antimicrobials for the control of bacteria pathogens. This strategy could be pursued whenever the primary and tertiary structures of a target are are conserved between eukaryotic and prokaryotes. A possible novel target fitting these parameters is choline kinase (ChoK), whose active site sequences are conserved (Figure 1) and whose tertiary structure (Figure 2) is maintained. Here, we describe why ChoK is a putative drug target by describing its role in the growth and pathogenesis of Gram-positive bacteria *S. pneumoniae* and the Gram-negative bacteria *H. influenzae*. Using *S. pneumoniae* as a model, we also present promising preliminary information that repurposing of drugs known to inhibit the human isoform of ChoK (hChoK), is a promising strategy for blocking the growth of *S. pneumoniae* cells and inhibiting the activity of the *S. pneumoniae* isoform of ChoK (sChok), with downstream physiological effects on the cell wall.

## Choline Kinase in Eurkaryotic Cells

Choline kinase catalyzes the conversion of choline (Cho) to phosphocholine (PCho) via the transfer of a phosphate group from ATP to Cho (Wittenberg and Kornberg, 1953). ChoK phosphorylation of Cho is the first step in the Kennedy pathway, which is responsible for the cell membrane component phosphatidylcholine, an important cell membrane element. Since it plays a role in the production of cell membrane components, ChoK functions as a mediator of cell growth and division of eukaryotic cells (Lacal, 2015). As a consequence, this enzyme is an important drug target for both tumor and parasite cells (Lacal, 2001; Zimmerman et al., 2013). Two isoforms of ChoK are found in mammalian cells, ChoK-α and Chok-β. ChoK-α is upregulated in many types of cancer cells including breast cancer (Shah et al., 2010), Pancreatic Ductal Adenocarcinoma (Mazarico et al., 2016), and many other types of cancer (Janardhan et al., 2006). Indeed hChok-α functions as a prognostic marker for cancer (Glunde and Bhujwalla, 2007). Many inhibitors have been developed that can block the activity of hChok-α as well as ChoKs from other eukaryotic cells. These molecules are known to block the proliferation of cancer cells (Lacal, 2008), to destroy eukaryotic parasites (Zimmerman et al., 2013), and to block autoimmune reactions (Guma et al., 2015). These inhibitors often function competitively by blocking choline from entering the active site (Hong et al., 2010), but there is a notable example of an hChoK-α known to bind an allosteric site in a uncompetitive fashion (Zimmerman et al., 2013; Kall et al., 2018). One of these, RSM-932A, has entered stage I clinical trials for patients with advanced tumors^1^, suggesting that the development of tumor treatments using ChoK is a well-developed avenue of research.

## Choline Kinase in Prokaryotic Cells

Bacterial pathogens such as *S. pneumoniae*, *H. influenzae*, *B. subtilis*, *Clostridium perfringens*, and *Clostridium botulinum* also express ChoK. In fact thousands of bacterial species are known carry a putative ChoK gene (see Figure 3 for Taxonomy), which suggests that the growth of any of these could be modulated using ChoK inhibitors. This strategy appears to be particularly promising in the case of the Gram-positive *S. pneumoniae* and the Gram-negative *H. influenzae*: two pathogens in which ChoK activity has been confirmed (Wang et al., 2015).

### Streptococcus pneumoniae

As in eukaryotic systems, sChoK phosphorylates choline into phosphocholine. Phosphocholine is important because it a precursor molecule that is utilized in the production of two types of teichoic acids, lipoteichoic acid (LTA) and cell wall teichoic acid (CTA) (Grundling and Schneewind, 2007; Whiting and Gillespie, 1996). In *S. pneumoniae*, both acids consist of the same types of polysaccharides but LTA is attached to a lipid and embedded in the cell membrane, and CTA is attached to the peptidoglycan molecules in the cell wall (Young et al., 2013). LTA is an important virulence factor and LTA production has been validated as a drug target (Ginsburg, 2002).

In the case of *S. pneumoniae* (Tomasz, 1967) choline is an essential nutrient.

The sChoK enzyme is an element of the pathway that mediates the production of teichoic acids via the intermediary CDP-choline. The elements of this pathway are expressed via the *lic* gene locus. The LicB gene expresses a Cho transporter, which collects Cho from the external environment. LicA codes for sChoK which phosphorylates Cho into PCho. LicC is a gene coding for cytidylyl transferase which converts PCho into CDP-choline. The LicD1 and LicD2 PCho transferases remove the PCho moieties from CDP-choline molecules and attach one PCho to each of the two N-acetylgalactosamine (GalNac) residues of the found on pre-teichoic acid glycan precursors (Denapaite et al., 2012). Aftward the precursors are assembled into pre-teichoic acid (Denapaite et al., 2012) (see Figure 4 for relevant pathway) and then embedded either into the membrane (LTA), or the cell wall (CTA). Interestingly, LidD2 knockouts of *S. pneumoniae* display limited virulence in mouse models and do not adhere as well to alveolar cells. This reduction in adherence is caused by limiting the number of PCho containing teichoic acids found on the cell surface (Zhang et al., 1999). Teichoic acid is then integrated into the cell membrane and wall by the teichoic acid flippase TacF. In order for the cell to function properly, Cho molecules must be incorporated into the cell surface. This is because Cho functions as an anchor for choline binding proteins (CBPs) that contain repeat amino acid sequences that recognize the choline head of LTAs. For example, the CBPs LytA and LytB are murein hydrolases that help remodel the cell wall during bacterial growth and cell division (Maestro and Sanz, 2016). LytA is responsible for deoxycholate induced autolysis while LytB is responsible for separating daughter cells at the end of cell division (Sanchezpuelles et al., 1990). Colonization of the nasopharynx is mediated by the choline binding adhesion protein CBPA which is a determinant of virulence (Rosenow et al., 1997). Many other CBPs are involved in colonization and even sepsis (Gosink et al., 2000). In summary, phosphocholine containing LTAs form a critical part of both the processes of cell division and invasion.

### Haemophilus influenzae

In the case of the Gram-negative *H. influenzae*, Cho is not a nutritional requirement, but this pathogen does acquire Cho from environmental sources (Weiser et al., 1997) and host cells (Fan et al., 2001), and produces PCho which, in turn, are used to decorate lipopolysaccharides (LPS). Modified LPS molecules mediate pathogen-host cell interactions allowing the pathogen to evade the host immune system (Young et al., 2013). Importantly, during colonization steps, the LicA gene is upregulated (High et al., 2015) PCho containing LPS molecules mimic the phosphatidylcholine of host cells, allowing it to evade anti-microbial peptides produced by host cells, such as cathelicidin LL-37/hCAP18 (Lysenko et al., 2000). PCho is also important for pathogenesis of *H. influenzae*, because this molecule mediates cell adhesion to the host PAF receptor (Weiser et al., 1997) in a step which leads to invasion the respiratory tract (Young et al., 2013). Binding to this receptor may lead to invasion of epithelial cells and sequestration from host clearance mechanisms (Swords et al., 2000). In addition PCho decoration of LTA has been show to reduce the binding of a bacteriocidal antibodies (Clark et al., 2012). In short, PCho also plays a role in the pathogenesis of *H. influenzae*.

## Hyphothesis

In light of the fact that the modification of cell surface molecules such as LTAs and LPS by PCho are critical to the pathogenesis of the *S. pneumonaie* and *H. influenzae*, it is probable that ChoK is a promising drug target for the treatment of these pathogens and likely many other species that produce ChoK. In the case of *S. pneumoniae* in particular it is likely that: (1) inhibitors designed to block the activity of hChoK will also block the activity of sChoK, and (2) inhibiting sChoK using these small molecules will inhibit the growth of *S. pneumoniae*.

## Drug Target Validation Using Schok as a Model

**FIGURE 1:**
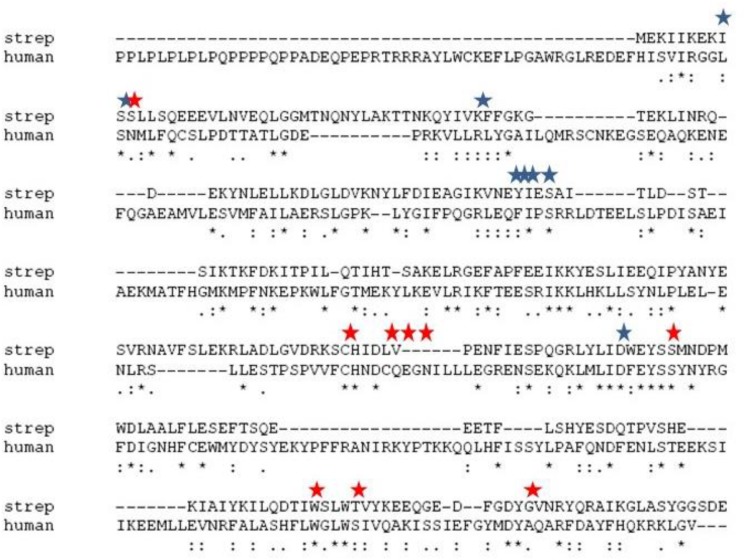
Alignment of the primary sequences of sChok and hChoK using ClustalW. The positions of the choline kinase binding residues of hChoK are shown in with a red star. The ATP binding site residues are shown with a blue star.

**FIGURE 2:**
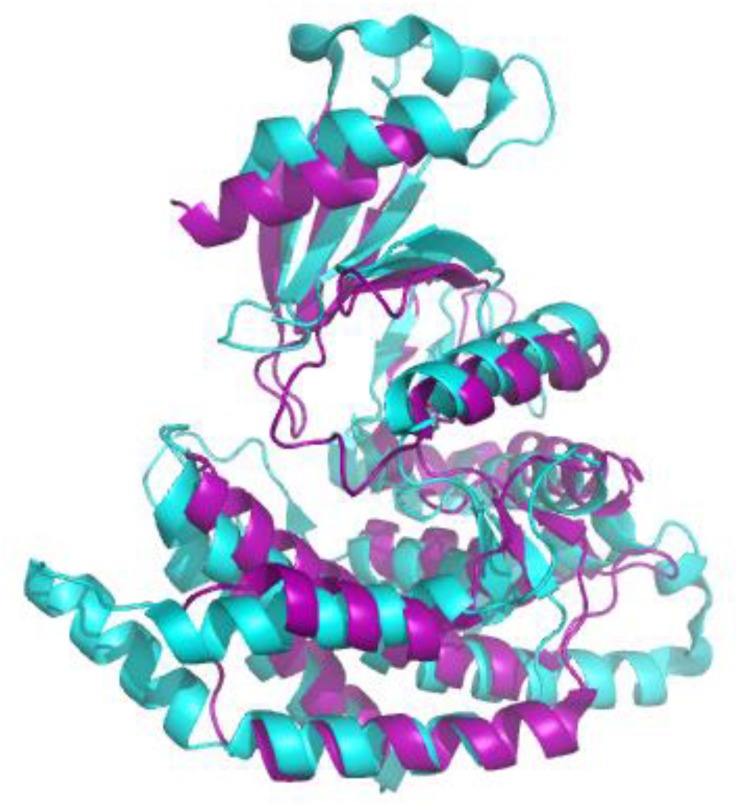
Alignment of the crystal structures of sChoK (purple. RCSB accession 4R77) and hChok (cyan, RCSB accession #2CKO). The basic N-terminal and C-terminal domains are shown to be generally conserved. Alignment and figure generation carried out with the PyMol Package.

We first evaluated the effects of the well characterized hChoK inhibitor Hemocholinium-3 (HC-3) on the sChoK activity and cell growth of *S. pneumoniae*. HC-3 was chosen as a test inhibitor because it is a known inhibitor of both cancerous (Lacal, 2001) and parasite cells (Zimmerman et al., 2013). HC-3 competes with the Cho substrate by directly displacing it from the active site of hChoK (15). The 3-dimensional structure of sChoK is conserved with respect to the human isoform (hChoK) which suggests available inhibitors for hChoK isoforms should be also be effective against sChoK (Figure 1; Wang et al., 2015). In addition, though the primary structure of sChoK is poorly conserved with respect to hChoK, residues of the catalytic site are highly conserved (see Figure 2), making HC-3 a likely positive candidate for sChoK inhibition.

**FIGURE 3:**
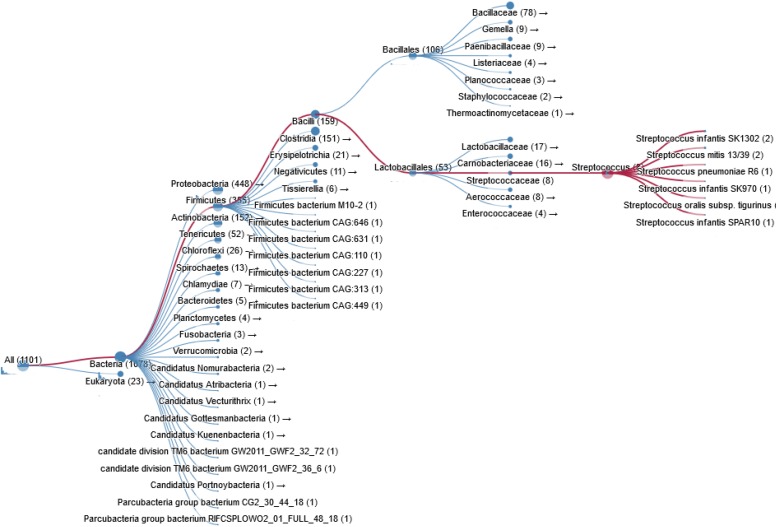
Taxonomy of prokaryotic species containing a ChoK gene that is highly conserved with respect to sChoK.

**FIGURE 4:**
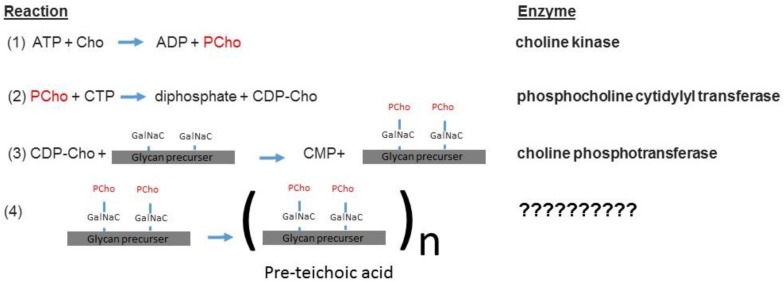
The sChok pathway and how it fits into the overall process of teichoic acid assembly (Denapaite et al., 2012).

Hemocholinium-3 was indeed shown to block cell division, reduce LTA production, distort the cell wall, and inhibit sChoK activity (Zimmerman and Ibrahim, 2017). However, the MIC and IC_50_ of HC-3 is high, 5400 μM and >2700 μM, respectively, therefore stronger sChoK inhibitors needed to be found.

Using a colorimetric system we developed for quantifying ChoK enzymatic activity in cells extracts (Zimmerman and Ibrahim, 2018), we measured the IC50s of two well characterized and efficient hChoK inhibitors (Ibrahim et al., 2018). These are MN58b (Hernandez-Alcoceba et al., 1997) and RSM-932A (Lacal and Campos, 2015) (see Table 1). In addition, we measured the minimum inhibitory concentration (MIC) of these drugs against cell cultures of the R6 strain *S. pneumoniae* (see Table 1). Both drugs were found to be several orders of magnitude stronger than HC-3, mimicking results from eukaryotic systems (Lacal, 2001; Zimmerman et al., 2013). In addition we found that these drugs dysregulated the production of LTA, distorted the cell wall, and affected cell sensitivity to deoxycholate induced autolysis (unpublished data). Altogether, these results suggested that the LTA pathway had been disrupted via inhibition of sChoK with strong downstream physiological consequences affecting cell growth.

**TABLE 1:** IC_50_ and MIC of HC-3, RSM-932A and MN58b.

**Drug**	**MIC**	**IC_50_**
HC-3	5400 μM	>2700 μM
RSM-932A	0.4 μM	0.5 μM
MN58b	10 μM	197 μM

## Perspectives

The results we have obtained reinforces the proposition that bacterial ChoKs are promising new drug targets that should be developed further, and that the same tools developed against eukaryotic ChoK activity can be exploited for use in pathogenic systems. In addition, the colorimetric system we have developed can efficiently be used to compare the relative strength of putative inhibitors of sChoK. The existence of ChoKs in other bacterial species where they are predicted remains to be confirmed. In addition, ChoK needs to be explored as target in species such as *H. influenzae* in which ChoK activity has been confirmed.

Despite the positive evidence we have seen with sChoK inhibition, in future research we will have to keep into account that any inhibitor capable of inhibiting ChoK, particularly choline analogs, may inhibit other CBPs such as the autolysin LytA (Maestro and Sanz, 2016). In our latest research we have started to account for this, and begun to distinguish between ChoK inhibitors that can block the autolytic process, such as HC-3 (Zimmerman et al., 2017) (presumably by interfering with the binding of autolysis to the choline head of LTA), and those that cannot, such as MN-58 and RSM-932A (unpublished data). However, while we do want to precise about possible multiple mechanisms of actions, having several targets is not a deficit for any putative sChoK inhibitor: a drug that disrupts more than one target of the choline metabolism pathway would be beneficial as multiple disruptions would make development of resistance to these antibiotics more difficult to achieve.

Another point to keep in mind is that, given the fact that ChoK and other CBPs are produced in many bacterial species, ChoK inhibitors is likely to affect many difference species including those found in the human microbiome. Therefore, in the future, we will need to take these effects into account in order to get a more nuanced view of the impact of these drugs on human health. Nevertheless, the same critique can and should be leveled at any new (and past) antibiotic ever developed or indeed of any medication, even aspirin (Obanla et al., 2016). Therefore, this critique by itself is not a reason to doubt the promise of pursuing the line of inquiry being proposed here because the possibility of developing any antibiotic that is specific to the bacterial species being targeted is remote indeed.

Another area of concern is that applying ChoK inhibitors to block an infection may lead to side effects by blocking the activity of hChoK. This concern is particularly valid for those inhibitors that have been developed to block hChoK, like MN-58b and RSM-932A. However, we deliberately chose the best characterized two hChoK inhibitors available for our initial studies. These characterizations include pre-clinical toxicity studies, that give us an idea of dosage limits (Lacal and Campos, 2015). Presumably, one would have to assess if these drugs are effective in animal and human models within the limits of toxicity. We know that these drugs are non-toxic to human primary cell lines and quite selective to tumor cells. Development of RSM-932A even reached phase I drug trials for treating tumors (see text footnote 1). Indeed these drugs are being explored for use in inflammatory disorders (Guma et al., 2015). What we are positing here is that the usefulness of these drugs could extend to use as antibiotics.

Of course the ultimate goal is to develop a drug that is specific to microbial ChoKs, and we are currently developing systems to screen libraries of putative inhibitors against human ChoK AND microbial ChoKs, to discover those that selectively inhibit the microbial homolog. The primary structures, apart from the active site, greatly differ (see Figure 1), therefore our expectation is that we will be able to find suitable candidates that are specific to microbial analogs. We are also searching for natural sources of microbial ChoK inhibitors from commonly consumed plant material. At this proof-of-concept stage, we tested the best developed candidates for hChoK and demonstrated that they can successfully inhibit sChoK with downstream physiological consequences for the *S. pneumoniae* cell that include inhibition of cell division.

To our knowledge, our studies are the first that demonstrate that ChoK could be used as a drug target in pathogens. What we describe here are the initial steps in the long journey toward establishing a novel line of antibiotics and antimicrobials for dealing with infections and food contamination using ChoK as the target of interest. The challenge of antibiotic resistance in bacterial pathogens must be faced with new tools such as these.

## Conclusion

It is imperative that new drug targets be discovered in *S. pneumoniae* and other pathogens as older antibiotics become obsolete through the evolution of resistant strains. ChoK shows promise as a new drug target that can be exploited for the development of a novel line of antibiotics.

## Author Contributions

All authors listed have made a substantial, direct and intellectual contribution to the work, and approved it for publication.

## Conflict of Interest Statement

The authors declare that the research was conducted in the absence of any commercial or financial relationships that could be construed as a potential conflict of interest.

## Footnotes

1 https://clinicaltrials.gov/ct2/show/NCT01215864
